# A Preclinical Evaluation of Alternative Synthetic Biomaterials for Fascial Defect Repair Using a Rat Abdominal Hernia Model

**DOI:** 10.1371/journal.pone.0050044

**Published:** 2012-11-20

**Authors:** Daniela Ulrich, Sharon L. Edwards, Jacinta F. White, Tommy Supit, John A. M. Ramshaw, Camden Lo, Anna Rosamilia, Jerome A. Werkmeister, Caroline E. Gargett

**Affiliations:** 1 Monash Institute of Medical Research, Clayton, Victoria, Australia; 2 Medical University Graz, Graz, Austria; 3 CSIRO Materials Science and Engineering, Clayton, Victoria, Australia; 4 Department of Obstetrics and Gynaecology and Monash Micro Imaging, Monash Medical Centre, Monash University, Clayton, Victoria, Australia; University of Minho, Portugal

## Abstract

**Introduction:**

Fascial defects are a common problem in the abdominal wall and in the vagina leading to hernia or pelvic organ prolapse that requires mesh enhancement to reduce operation failure. However, the long-term outcome of synthetic mesh surgery may be unsatisfactory due to post-surgical complications. We hypothesized that mesh fabricated from alternative synthetic polymers may evoke a different tissue response, and provide more appropriate mechanical properties for hernia repair. Our aim was to compare the in vivo biocompatibility of new synthetic meshes with a commercial mesh.

**Methods:**

We have fabricated 3 new warp-knitted synthetic meshes from different polymers with different tensile properties polyetheretherketone (PEEK), polyamide (PA) and a composite, gelatin coated PA (PA+G). The rat abdominal hernia model was used to implant the meshes (25×35 mm, n = 24/ group). After 7, 30, 60, 90 days tissues were explanted for immunohistochemical assessment of foreign body reaction and tissue integration, using CD31, CD45, CD68, alpha-SMA antibodies. The images were analysed using an image analysis software program. Biomechanical properties were uniaxially evaluated using an Instron Tensile® Tester.

**Results:**

This study showed that the new meshes induced complex differences in the type of foreign body reaction over the time course of implantation. The PA, and particularly the composite PA+G meshes, evoked a milder early inflammatory response, and macrophages were apparent throughout the time course. Our meshes led to better tissue integration and new collagen deposition, particularly with the PA+G meshes, as well as greater and sustained neovascularisation compared with the PP meshes.

**Conclusion:**

PA, PA+G and PEEK appear to be well tolerated and are biocompatible, evoking an overlapping and different host tissue response with time that might convey mechanical variations in the healing tissue. These new meshes comprising different polymers may provide an alternative option for future treatment of fascial defects.

## Introduction

Incisional ventral hernias occur in up to 49% of patients after abdominal surgery for trauma, infection, herniation or surgical resection [1]–[3]. Similarly, hernias in the female lower urogenital tract known as pelvic organ prolapse (POP) occur in up to 25% of women with childbirth, ageing and obesity being the most common causes [4]. Recurrence rates with tissue approximation procedures occur in up to 17% for abdominal hernias [5] and 30% for POP repair [6], respectively, which has led to the implantation of synthetic meshes to improve surgical outcomes.

Polypropylene (PP) mesh is the most commonly used mesh, and is knitted from monofilament yarn to a relatively large pore size, in order to allow tissue ingrowth [7]. Synthetic non-degradable meshes are used to provide a permanent solution and are believed to work by inducing an inflammatory foreign body reaction, resulting in fibrosis that provides strength, albeit not necessarily mechanically optimal, to the weakened support structures [8], [9]. However, the presence of an inflammatory response may reflect poor tissue biocompatibility, depending on the extent and type of response. It is also primarily responsible for the significant complications arising from the use of synthetic meshes reported in up to 42%, including mesh contraction, infection, pain, and exposure of mesh [10], [11].

With PP mesh dominating the market, we believe that mesh manufactured from alternative synthetic polymers may evoke a different biological response, and provide more appropriate mechanical properties for hernia repair. PA and PEEK polymers were chosen as alternative polymers to PP, due to differences in yarn tensile properties. Gelatin coating was also used to further improve tissue integration [12], [13].Gelatin works as a carrier for delivering large number of cells and can be adapted as a basis for cell based therapies in the future [14].

The aim of this study was to compare the *in vivo* host tissue response of synthetic meshes fabricated from alternative polymers and a composite mesh coated with gelatin, with a clinical PP mesh, using a rat abdominal hernia model as a preclinical model for hernia repair surgery.

## Materials and Methods

Polyetheretherketone (PEEK) and polyamide (PA) meshes were warp knitted to the same pattern using 100 µm PEEK (Invibio; UK) and PA (Ri-Thai International; Taiwan) monofilament yarns. Meshes were knitted to possess pore sizes ranging 1310 to 1487 µm and weights ranging 69 to 85 g/m^2^. A clinical PP mesh (Polyform ™; Boston Scientific; USA), of similar knit pattern, filament diameter and pore size, with a weight of 42 g/m^2^ [15], was also used in the study. Additionally, fabricated PA mesh was coated with gelatin (PA+G). Briefly, PA mesh was immersed in a 0.2 µm filter sterilised solution of 12% porcine gelatin (Type A, 300 bloom; Sigma; USA), in water. Once fully wetted the mesh was placed onto ice-cold 2% glutaraldehyde (Sigma) in PBS for 8 minutes on either side. All the subsequent steps were for 15 minutes duration and at room temperature. The mesh was washed, placed into 2% w/v glycine (Merck; Australia) in water, washed, placed in 2% v/v H_2_O_2_ (Merck) in water, washed, then 4% w/v glycerol (Merck) in water. Finally, the mesh was air-dried overnight. All mesh samples were gamma ray irradiated at 25 kGy prior to implantation.

### Animals

The experimental procedures and rat husbandry were approved by the Monash Medical Centre Animal Ethics Committee A (2009/50). Sprague Dawley rats were housed in the animal house of Monash Animal Service facilities in compliance with the National Health and Medical Research guidelines for the care and use of laboratory animals. The rats were provided with food and water *ad libitum* and were kept under controlled environmental conditions at 20°C and a 12 h day/night cycle.

### Surgical Procedure

125 female rats were divided into 5 experimental groups (24 rats/group) and were implanted with one of 4 mesh types; PA, PA+G, PEEK, or PP. A sham operation was used as a negative control as described below. The rats were anaesthetised with 2.5% Isoflurane® and analgesia was provided with Carprofen (5 mg/kg bodyweight). The abdomen was shaved and disinfected and covered with sterile drapes. A longitudinal 3 cm skin incision was performed in the lower abdomen and a full thickness fascial defect of the abdominal wall, 20×30 mm, was created [16] (Fig. S1A) . Before implantation, mesh thickness was measured with a calliper. Each mesh (25 mm×35 mm) was implanted using the overlay technique (Fig. S1A) and sutured with PP sutures as previously described [16], [17] (Fig. S1C). Rats in the control group were sham-operated without mesh implantation. A 25×35 mm full-thickness abdominal wall defect was created on the right lateral side of the midline. A muscle flap with preserved vascularisation from the contralateral side was created, rotated 45° clockwise and sutured with 3/0 Surgipro™ II (polypropylene thread) to cover the initial defect (Fig. S1B). Skin closure in all groups was performed by intracutaneous continuous suture with 3/0 Vicryl* Plus (polyglactin thread).

Following recovery, the animals were monitored daily until they were sacrificed at 7, 30, 60 and 90 days (n = 6/ group/ timepoint). Each rat was euthanized in a CO_2_ chamber. The adhesion area of the mesh on the intestinal side was recorded and calculated as a percentage of total mesh area. Adhesion tenacity to the intestinal wall was also recorded as previously described [18]. Adhesion scores were graded from 1–4 with 4 being the most adhesive. The mesh was dissected with a 5 mm border of adjacent tissue and divided into three parts; for biomechanical analysis, histology and immunohistochemistry, using the same pattern of dissection for each mesh. Adhesive organs to the mesh were peeled off manually. The meshes used for biomechanical analysis were stored at −20 degrees prior to testing. Additionally, explant dimensions (length, width and thickness) were measured with a calliper at 3 random regions and the mean recorded. Mesh contraction was calculated by dividing the mesh size after explantation by the original mesh size.

### Biomechanical Analysis

The frozen samples were thawed overnight at 4 degrees and kept moist during testing. Day 7 and day 90 explants and tissue from the control rats were uniaxially tensile tested using an Instron® Tensile Tester (Instron Corp; USA) and a 5 kN load cell. Samples (typically n = 10), with dimensions of 4×25 mm, were cut in the explant cross direction and tested using a 14 mm gauge length (noted as the initial length). Samples were pre-loaded to 100 mN at an elongation rate of 14 mm/min to remove any sample slack. Subsequent testing was conducted at an elongation rate of 30 mm/min to break. Average load-elongation curves were plotted for each mesh type and the control (failure region not included in curve) from the data obtained. Tensile strain (%) was calculated by dividing mesh elongation by the initial mesh length and multiplying by 100. Mesh stiffness is represented by the slope of the curve, with a steeper curve indicating a stiffer mesh.

### Histology

The explanted tissue for histological analysis was fixed in 4% w/v paraformaldehyde (PFA) for 24 h, then embedded in paraffin and sectioned into 5 µm thick sections. After dewaxing and rehydrating in graded alcohols, sections were stained with haematoxylin and eosin (H+E) or Sirius red F3B (0.1 g/100 ml saturated aqueous picric acid) (Sigma Aldrich®, St. Louis, MO USA) for one hour at room temperature (RT).

### Immunohistochemistry (IHC)

To examine for tissue macrophages, a monoclonal anti rat CD68 antibody (details in Table 1) was used. Sections underwent dewaxing, rehydrating in graded alcohols. Antigen retrieval using citric acid buffer (0.1 M, pH 6.0) was done by microwaving for 5 minutes on high power. After cooling to RT and washes in PBS, endogenous peroxidase was quenched with 3% v/v H_2_O_2_ followed by protein blocking (Protein Block serum free, Dako®, Glostrup, Denmark) for 30 minutes at RT. The primary CD68 antibody and isotype controls (mouse IgG_1_) were incubated overnight at 4°C, sections washed and the secondary Streptavidin HRP-conjugated antibody was applied for 30 minutes at RT, respectively (Table 1). 3,3′-Diaminobenzidine (Sigma-Aldrich®, St. Louis, MO, USA) was used as a chromogen, counterstaining was with haematoxylin. The slides were dehydrated in graded alcohols and mounted with DPX mounting medium.

**Table 1:** Details of antibodies used for immunohistochemistry.

Primary antibody	Clone	Dilution	Isotype	supplier	Secondary antibody	Dilution	supplier
α smoothmuscle actin	1A4	1∶400	Mouse IgG2a	Dako®, Glostrup. Denmark	Alexa fluor 488 goatanti-mouse IgG	1∶200	Invitrogen®, Mulgrave, VIC, Australia
CD 31PECAM-1	TLD-3A12	1∶200	Mouse IgG1	BD Pharmingen®NJ, USA	Alexa fluor 568 donkeyanti-mouse IgG (H+L)	1∶200	Invitrogen® Mulgrave, VIC, Australia
CD 45Leukocyte commonantigen	OX-1	1∶200	Mouse IgG1	BDPharmingen®NJ, USA	Alexa fluor 568donkey anti-mouseIgG (H+L)	1∶200	Invitrogen® Mulgrave, VIC, Australia
CD 68/MCA341R	ED 1	1∶500	Mouse IgG1	AbD Serotec®, Oxford, UK	EnVison+System- HRP Labelled Polymer (anti-mouse)	Ready to use	Dako®, CA, USA

### Immunofluorescence

4% w/v PFA fixed, paraffin embedded tissues were used for immunofluorescence analysis and 5 µm sections dewaxed, dehydrated, blocked and washed as above. Sections were incubated with the primary monoclonal anti-alpha smooth muscle actin (αSMA) antibody for one hour at 37°C (Table 1) to label smooth muscle cells, myofibroblasts and myoepithelial cells. Mouse IgG_2a_ isotype was used for the negative control and applied at the same concentration. Bound antibodies were detected with Alexa-Fluor-488- conjugated secondary antibody (Table 1) for 30 minutes at RT after 3 washes in PBS. Nuclei were stained with Hoechst 33258. The slides were mounted with fluorescent mounting medium (Dako®, Glostrup, Denmark).

CD31 and CD45 immunofluorescence was used to visualise endothelial cells and leukocytes, respectively (Table 1). Tissue for frozen sections was snap frozen in optimal compound tissue solution (OCT, Tissue-Tek, Miles, IN, USA). The frozen sections cut (5 µm) were thawed at RT, fixed in ice cold acetone for 10 minutes followed by protein block (Dako®, Glostrup, Denmark) for 30 minutes at RT. After three washes in PBS, primary antibodies were incubated for 1hr at 37°C and with Alexa-Fluor-568- conjugated secondary antibody (Table 1) for 30 minutes at RT, respectively. The slides were mounted as above.

### Histomorphometric Analysis

**Figure 1:**
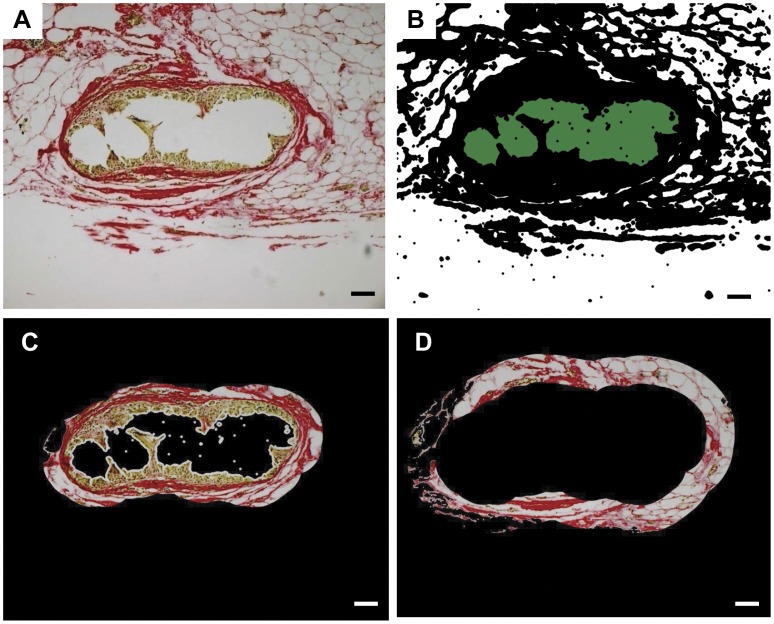
Image analysis using Metamorph®. Software. A. Original image showing Sirius red staining of tissue around a central filament bundle, scale bar 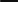 100 µm. B. binarized image with mesh recognition. C. First 50 µm increment surrounding filament bundle. D. Second 50 µm increment (50–100 µm) surrounding filament bundle.

Four images were taken per section stained with Sirius red or immunostained with CD68, αSMA, CD31 or CD45 for each explant at each timepoint using a Leica® DMR Fluorescence Microscope at 20X 0.6NA magnification and at 10X 0.3NA for H+E. Images of the 4 most central mesh filaments were captured (Fig. S2A, B, Fig. 1A), to achieve consistency and overcome bias, and to avoid any foreign body reaction associated with anchoring sutures. The images were analysed using the image analysis software, Metamorph® (v7.7 Molecular Devices, LLC, CA, USA). The software was programmed to identify individual mesh filaments (Fig. 1B) and trace contoured concentric area bands around filament bundles in 50 µm increments (Fig. 1C, D). The positive signal area (pixels) for each shape was recorded and divided by the total tissue area examined (Fig. 1B). Neotissue formation (as indicated by Sirius, αSMA and CD31) was analyzed using the whole image, assessing the entire surrounding tissue. Foreign body cells were assessed close to the mesh filaments.

### Statistics

GraphPad Prism 5 was used for statistical analysis. Results are reported as mean ± SEM for each experimental group (n = 6 animals/ group/ timepoint). Since the data was not normally distributed (D’Agostino & Pearson omnibus normality test), non-parametric analysis using Kruskal – Wallis ANOVA, were undertaken to assess differences between timepoints for the various meshes, followed by Bonferroni correction for pairwise comparisons. P values <0.05 were considered as statistically significant. For statistical analysis of load-elongation curves, 95% confidence intervals were plotted as error bars to determine whether the differences were significant at the p <0.05 level of significance.

## Results

### Macroscopic Tissue Compatibility of New Meshes

Two new meshes, warp-knitted from PA and PEEK monofilaments in a similar pattern as commercially available PP mesh, were examined for tissue biocompatibility using a standard rat abdominal hernia model. In addition, a gelatin-coated PA mesh was studied, since this composite could be adapted for delivering cells in future experiments. Of the 125 animals operated, all had a normal post-operative recovery. Five rats developed wound infection or wound dehiscence with no differences between the mesh groups, necessitating killing these animals according to local ethics committee regulations. Therefore a total number of 120 animals (6 per group at each timepoint) were included for analysis.

**Figure 2:**
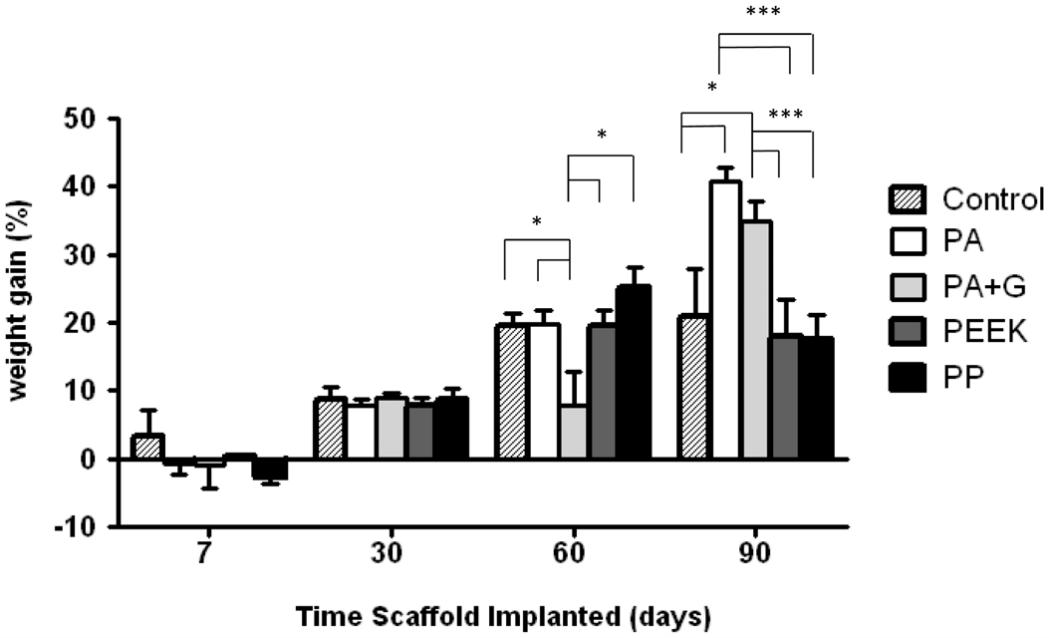
Body weight gain of rats implanted with different synthetic meshes: PA, PA+G, PEEK or PP and controls. Data are mean ± SEM of n = 6 animals/ group, * p<0.05, *** p<0.001. PA, polyamide; PA+G, polyamide gelatin composite; PEEK, polyetheretherketone; PP, polypropylene.

All mesh types were well tolerated. Rats gained weight 30 days after operation and weight gain increased significantly in rats implanted with PA, PEEK and PP meshes at 60 days (p<0.05) (Fig. 2). At 90 days, rats implanted with PA and PA+G meshes showed significantly greater weight gain compared to control rats, and rats implanted with PEEK and PP meshes (p<0.001). Ninety days after surgery, weight gain was significantly higher in all groups compared to 7 days (pairwise comparisons p<0.0001).

**Table 2:** Adhesion scores of rats implanted with meshes.

Time (days)	PA	PA+G	PEEK	PP
7	1.0±0.0	1.0±0.0	1.0±0.0	1.0±0.0
30	1.0±0.0	1.0±0.0	1.0±0.0	1.0±0.0
60	2.0±0.3	2.0±0.0	1.8±0.2	2.3±0.2
90	1.3±0.2a,b	1.8±0.2	2.0±0.0	2.0±0.0

Data are mean ± SEM.

a p<0.05 compared to PA+G.

b p<0.001 compared to PEEK and PP.

**Table 3:** Percentage adhesion area of meshes implanted into abdominal fascial wound in rats.

Time (days)	PA	PA+G	PEEK	PP
7	88±4	73±14	86±5	80±9
30	72±10^a^	40±4	40±8	73±8^b^
60	48±8	42±8	43±8	63±7
90	52±8	53±4	55±6	72±4

Data are mean ± SEM.

a p<0.05 compared to PA+G and PEEK.

b p<0.05 compared to PA+G and PEEK.

**Table 4:** Percentage mesh contraction in rats implanted with meshes.

Timepoints	PA	PA+G	PEEK	PP	
7	17.8±2.0	7.1±1.9	19.7±5.1	8.7±2.5	
30	29.9±7.0^a^	21.1±5.0	10.9±4.3	26.7±3.0^b^	
60	13.0±5.0	9.2±2.4	11.2±2.4	14.6±2.0	
90	14.7±5.4	9.0±4.3	19.4±3.0	15.1±2.8	

Data are presented as percentage reduction in size from original implanted mesh as mean ± SEM.

a p<0.01 compared to PEEK.

b p<0.05 compared to PEEK.

* denotes gelatin around PA filaments.

At the time of necropsy (Fig. S1D, E), intraperitoneal adhesions were noted mainly with the ileum and the omentum over the whole implant surface for all materials as well as to the sutures in all mesh groups. The adhesion scores are shown in Table 2. No differences were observed at 7 and 30 days between the mesh groups. PA meshes showed a significantly lower adhesion score compared to PA+G (p<0.05), PEEK and PP (p<0.001) at 90 days. Adhesion scores for PA+G, PEEK and PP meshes were significantly increased at 60 and 90 days compared to the earlier timepoints for the same mesh type (p<0.05).

**Figure 3:**
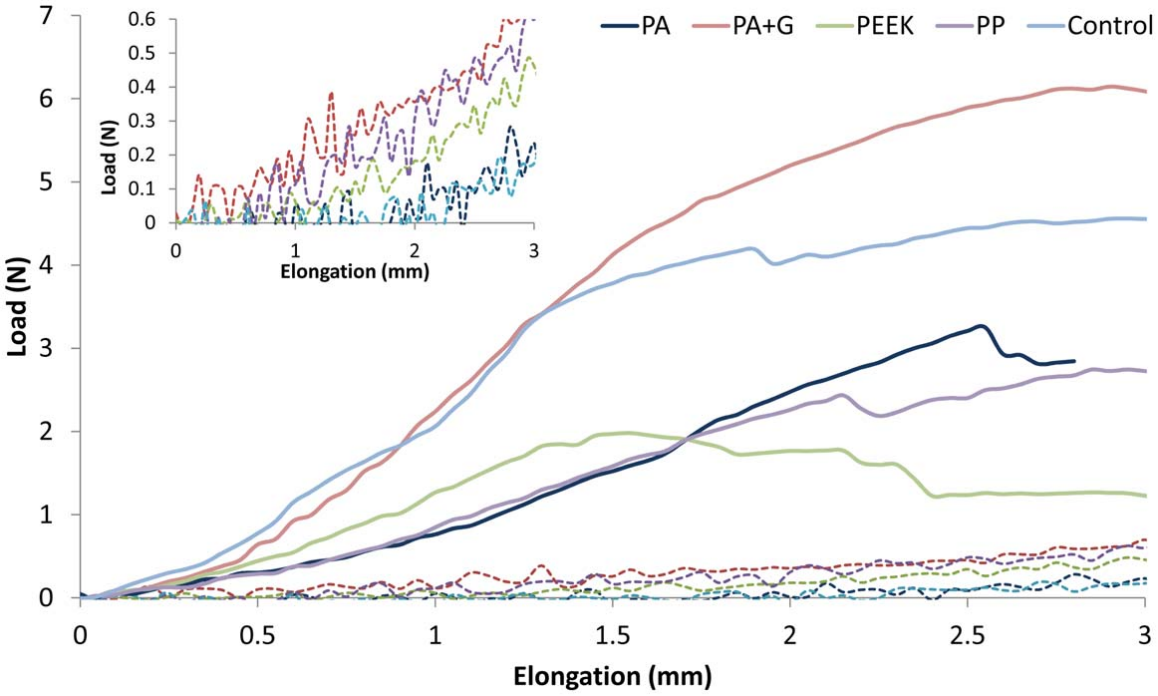
Biomechanical properties of explanted mesh types and control on day 7 (- - -) and day 90 (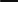). Failure region of graph has not been included. Average explant stiffness is indicated by the slope of the curve, with stiffer materials possessing a steeper gradient. Inset is an enlargement of day 7 meshes.

While all meshes displayed the greatest adhesion area at 7 days (range 73 to 88%, Table 3), the strength of adhesion was low (adhesion score of 1.0, Table 2). The extent of adhesion was lowest with the PA+G mesh at day 7 without statistically significant differences (Table 3). At 30 days, the extent of adhesion with the PA+G and PEEK meshes was significantly less than observed with PA and PP (p<0.05), with no significant difference by 60 and 90 days. All meshes reached the lowest adhesion area at 60 days, with no further reduction at 90 days, although none of these differences were significant.

**Figure 4:**
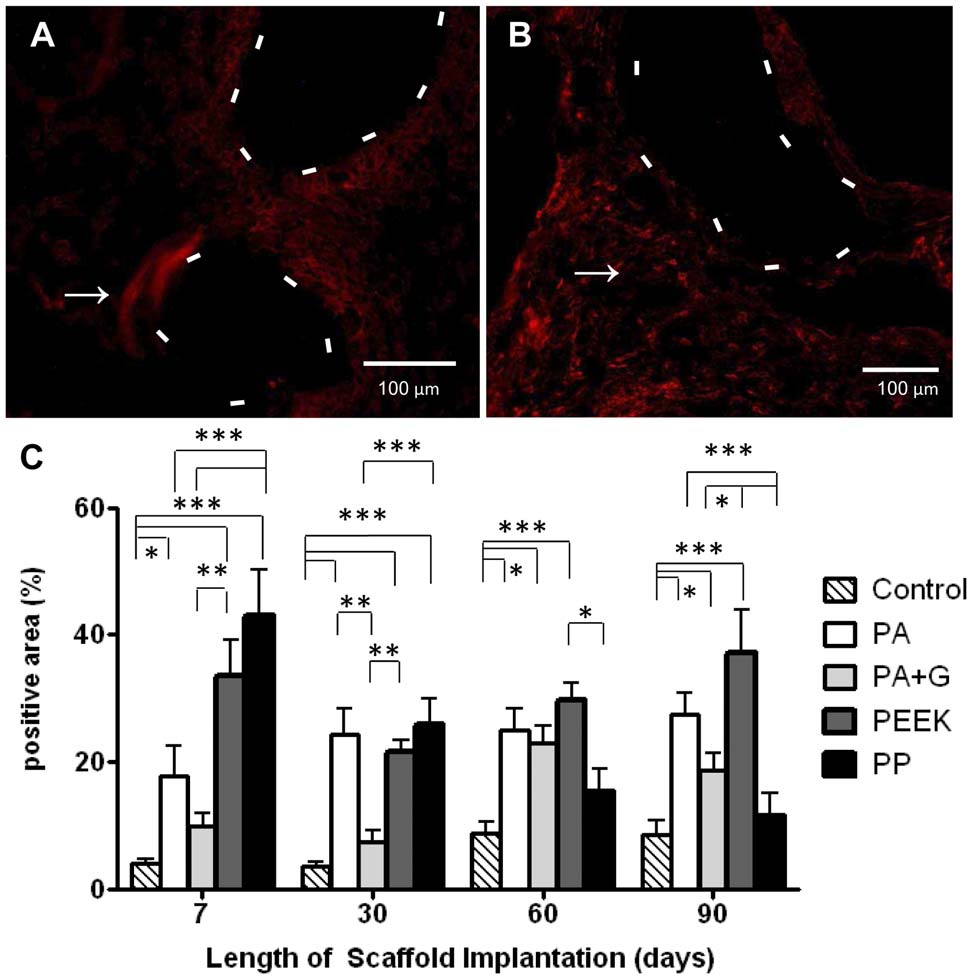
CD45 immunostaining in rats implanted with synthetic mesh. A. PA after 7 days (- - - line indicates mesh filament) and B. After 90 days implantation (arrow indicates a representative CD45 positive cell). C. Percentage CD45 positive area within the 50 µm radius of central mesh filament bundles. Data are mean ± SEM of n = 6 animals/ group *: p<0.05, **: p<0.01 Scale bar, 100 µm.

Folding of the meshes, which contributes to apparent mesh shrinkage, was occasionally observed, with the highest incidence seen in the PA and PP groups. Folding may have arisen from poor suturing but could also be due to extensive tissue ingrowth. These meshes were included in the analysis of mesh contraction. Contraction was significantly greater at 30 days compared to 7 days for PA+G and PP meshes; no significant differences in mesh contraction for PA and PEEK meshes were observed between these timepoints (Table 4). PEEK meshes shrank significantly less than PA and PP meshes at 30 days; however no significant difference was observed at 60 or 90 days (range at 90 days 9 to 19.4%, Table 4).

**Figure 5:**
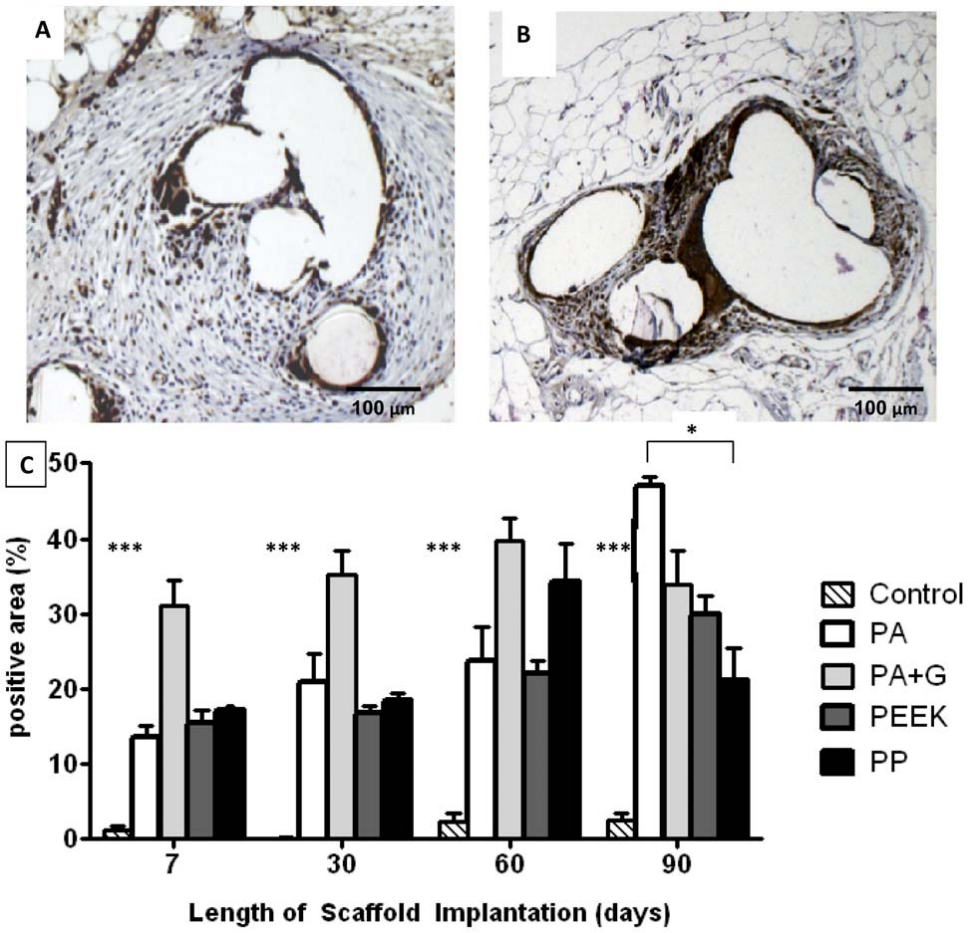
CD68 immunostaining in rats implanted with synthetic mesh. A. PA after 7 days and B. after 90 days implantation (- - - line around mesh filament). Brown staining indicates CD68^+^ macrophages. C. Percentage CD68 positive area within 50 µm radius of central mesh filament bundle. Data are mean ± SEM of n = 6 animals/ group, * p<0.05; *** p<0.001. Scale bar, 100 µm.

### Biomechanical Properties

The load-elongation curves for the day 7 and day 90 explants are shown in Figure 3 and in Fig. S3. The mechanical properties of meshes prior to implantation were not significantly different to those measured after 7 days implantation. Comparable load-elongation curves, with similar levels of stiffness, were observed for explanted mesh types and the control tissue on day 7; error bars suggest that curves were not significantly different from each other (p>0.05). The curves of day 7 meshes suggest some bilinearality, with a linear region from 1.7 mm elongation (Fig. 3 inset), however this is less obvious than for the day 90 meshes (which have distinct toe and linear regions). The load-elongation curves of all meshes explanted at day 90 were significantly (p<0.05) different to their day 7 counterparts exceeding 0.9 mm elongation (6.4% strain), and generally had increased stiffness (with steeper slopes) in the linear region of the curves (generally exceeding 0.6 mm elongation: 4.2% strain) (Fig. 3). At day 90, the PA+G explanted meshes were the stiffest of all the explanted meshes, and comparable to the control tissue explants, although this was only significant (p<0.05) at elongations exceeding 2.0 mm (14% strain).

**Figure 6:**
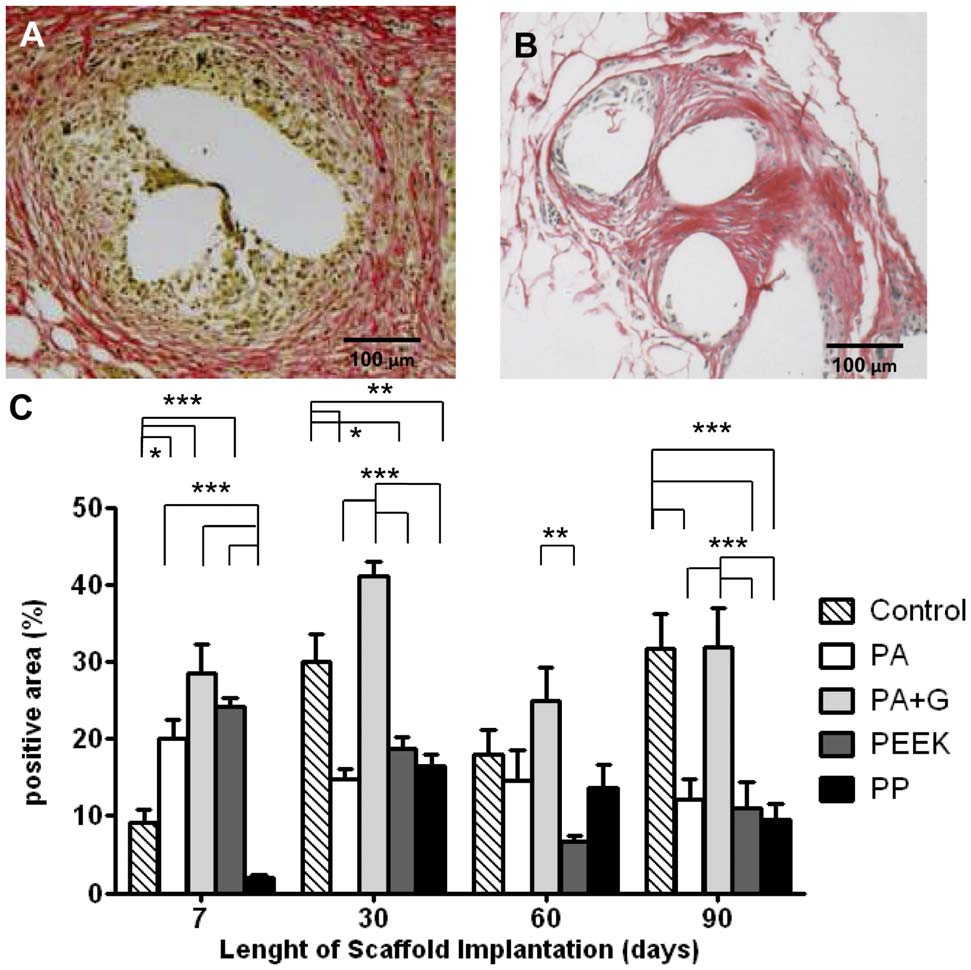
Sirius red staining of collagen fibres in rats implanted with synthetic mesh. A. PEEK after 7 days and B. After 90 days implantation. C. Percentage Sirius red stained area within 50 µm radius of central mesh filament bundle. Data are mean ± SEM of n = 6 animals/ group *: p<0.05; **: p<0.01, ***: p<0.001, Scale bar, 100 µm.

### Tissue Biocompatibility of Meshes

We examined the microscopic changes induced by the implanted mesh materials using histological stains and immunohistochemistry. As shown in Fig. S2A and B there was substantial cell and tissue formation around the mesh filaments at 7 days. At 90 days some encapsulation tissue was still apparent around all filament bundles. PA+G meshes had a greater tissue and cell response around the mesh filaments at 90 days compared to the other mesh types. No microscopic differences were apparent in the control group between 7 and 90 days.

**Figure 7:**
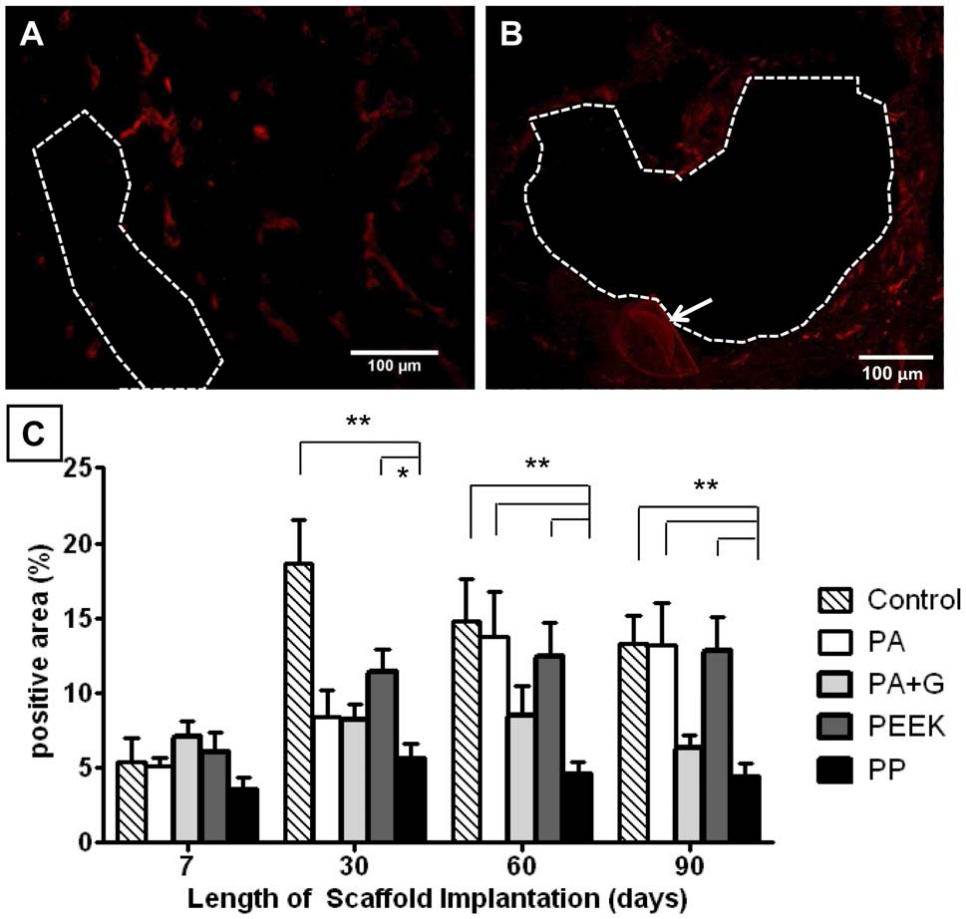
CD31 immunostaining in rats implanted with synthetic mesh. A. PA+G after 7 days and B. After 90 days implantation (- - - line around mesh filament). C: Percentage positive CD31 area within 50 µm radius of mesh. Data are mean ± SEM of n = 6 animals/ group, *p<0.05, **p<0.01.

**Figure 8:**
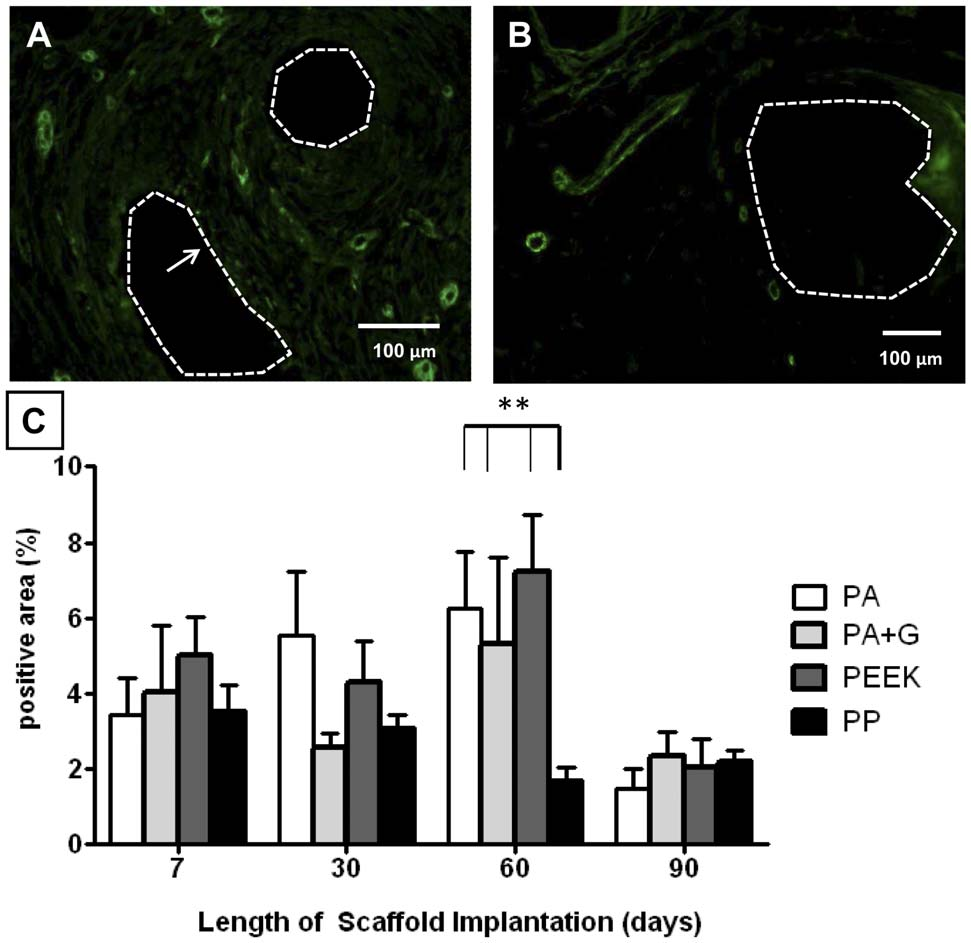
αSMA staining in rats implanted with synthetic mesh. A. PEEK after 7 days and B. after 90 days after implantation (dotted line around mesh filament, arrow indicates vessel). C. Percentage of positive αSMA staining. Data are mean ± SEM of n = 6 animals/ group, **: p<0.01. Scale bar, 100 µm.

To quantify this tissue response, image analysis was undertaken to examine the gradient of positive staining in 50 µm increments from the central mesh filaments. A diminishing gradient, with a significant decrease, in CD45 and CD68 immunostaining was observed with increasing distance from the filaments (Fig. S4A). We therefore studied the immediate area surrounding the mesh (within 50 µm). No continuous gradient was observed for the other stains (Sirius red, αSMA and CD31) (Fig. S4B), which were analysed using the entire micrograph.

### Inflammatory Foreign Body Reaction

The inflammatory response to the implanted meshes was evaluated by examining the extent of CD45^+^ leukocyte distribution in and around the meshes. CD45 is expressed on all hematopoietic cells except erythrocytes. Larger numbers of leukocytes (red fluorescence) were observed around the meshes (Fig. 4A) at all time points (Fig. 4C), compared to controls where no meshes were present (Fig. 4C). There were significantly fewer CD45 positive cells at 7 and 30 days in the control and PA+G groups compared to PA, PEEK and PP (p<0.001) (Fig. 4C). At 60 days PA, PA+G and PEEK attracted significantly more leukocytes than the control (p<0.05), with higher positive staining for PEEK, compared to PP (p<0.05). At 90 days, the new meshes showed greater leukocyte infiltration than the control and PP groups (p<0.001) (Fig. 4C).

To assess macrophage involvement we used the CD68 antibody (Fig. 5). The kinetics of macrophage infiltration varied between mesh types. For example, for the PA mesh, macrophages significantly increased over time from day 7 (Fig. 5A) to day 90 (Fig. 5C) (p<0.001). Significantly more macrophages were observed around the filaments for all mesh types than in the control group (p<0.001), at all timepoints (Fig. 5C). At 90 days PA meshes were surrounded by significantly more macrophages than PP (p<0.05) (Fig. 5C).

### Collagen Deposition

Collagen deposition was assessed using Sirius red staining which shows the collagen as red stained material in bright-field microscopy (Figure 6A, B). On day 7 PEEK meshes showed pronounced Sirius red staining loosely distributed around a dense cellular infiltrate (Fig. 6A). At day 90, the cellular infiltrate was less and collagen levels were reduced but more localised around the filaments of the porous mesh (Fig. 6B). At 7 days, a significantly lower collagen content was observed in the control and PP groups compared to PA, PA+G and PEEK meshes (p<0.001) (Fig. 6C). At 30 days, PA+G mesh induced the highest collagen deposition which was significantly greater than PA, PEEK and PP meshes (p<0.001), and comparable to control animals. At 90 days PA, PEEK and PP meshes were all significantly lower in collagen content (p<0.001) than the control, whilst the PA+G mesh was significantly higher, and comparable to the control (Fig. 6C). Compared to 7 days, collagen content decreased significantly in both PA and PEEK meshes (p<0.05) at 90 days, whereas collagen increased significantly in PP meshes (p<0.05).

### Vascularisation

The development of vascular structures in the explanted meshes was assessed using the marker CD31 which is expressed on endothelial cells, platelets and subsets of leukocytes. It predominantly stains endothelial cells on small and large blood vessels. Similar to all mesh types, the PA +G meshes were vascularised by day 7 (Fig. 7A), with vessels present at each time point, until day 90 (Fig. 7B). The positive area for CD31 (Fig. 7C) was similar between all mesh groups, and comparable with control animals, on day 7. On day 30 the control group and PEEK meshes showed significantly more positive staining than the PP meshes (p<0.01, p<0.05 respectively) (Fig. 7C). Vascularisation of PA and PEEK mesh types, and control animals was significantly higher than in PP meshes, at 60 and 90 days (p<0.01) (Fig. 7C). The extent of vascularisation increased with time for the PA meshes, and was significantly greater at 60 and 90 days compared to day 7 and day 30 (p<0.05). Vascularisation in the PEEK and PA+G meshes was similar at the different timepoints, as were the PP meshes, which had the least CD31 staining overall. To assess larger (stabilized) vessels having a smooth muscle cell coat, αSMA staining was performed. Figure 8A shows a large number of immunostained myofibroblasts in the PEEK meshes on day 7, which were similarly observed in all mesh types at 7 and 30 days. Larger vessels were seen at 90 days, for example in PEEK meshes (Fig. 8B). All meshes induced similar levels of myofibroblast differentiation and smooth muscle cells at 7 and 30 days (Fig. 8C). At 60 days, PA, PA+G and PEEK meshes had significantly more αSMA staining compared to PP meshes (P<0.01). At 90 days, all mesh types had a significantly lower positive signal compared to the other time points with no differences between the mesh groups.

## Discussion

In this study we compared the *in vivo* biocompatibility of alternative synthetic meshes with a clinical PP mesh, and a tissue control, by investigating biomechanics, collagen deposition, vascularisation and foreign body tissue reaction. Synthetic mesh, manufactured from alternative polymers to a clinical PP mesh, were warp knitted to a similar pattern, using yarn of the same diameter. This ensured that mesh pores were similar in size. Any differences in the host biological response were, therefore, a result of the polymer alone or of the coating. We studied the effect of gelatin coating only in the PA mesh as the tissue response to this composite mesh deemed to be representative for all polymers. This study showed that our new meshes induced a typical foreign body reaction over the time course, largely comparable with the PP meshes, but that there were differences with the extent and type of responses associated with inflammation, macrophage infiltration, collagen deposition and neovascularisation over the 90 day period.

In general, the PA+G meshes showed the least early CD45+ve inflammatory response at 7 and 30 days suggesting that the coating acts like a biofilm, while the PEEK and PP meshes were associated with a more acute inflammation that persisted with the PEEK meshes and subsided with the PP meshes over time. Nonetheless, the macrophage CD68 response depicted a general acute response to all implants. Generally, after implantation of a mesh an acute inflammatory reaction occurs, with macrophages dominating the acute phase at 7 days [16]. In our study, the inflammatory response to the foreign materials showed typical temporal differences. The PA+G meshes had notably more macrophages than the other mesh types at Day 7. Given that these mesh types had the lowest CD45 response at the same time point, it is likely that the major acute response here is due to macrophages. The PP response is similar to that described by Spelzini et al who also showed a lower foreign body reaction at 90 days around the PP implant compared to silk implants [19]. Apart from its role in acute inflammation and early vascularisation, macrophages also have a critical role in the subsequent chronic phase of the host response. In particular, macrophages can potentially differentiate towards two pathways, one leading to an immediate and/or persistent inflammation, the other leading to a constructive remodelling and new tissue generation [20]. This M1/M2 macrophage polarisation has not been studied in this current study. In the case with the PA+G meshes, the gelatin was found to be degrading at the later stages of implantation, associated with more available porous openings allowing fibroblast infiltration and new collagen deposition. It is likely, although speculative, that in these meshes immunoregulatory M2 macrophages are the major type of cell.

Consistent with this, our meshes led to better tissue integration (collagen deposition for PA+G on day 90) and neovascularisation at day 60 compared with the PP meshes. All mesh types were generally well tolerated, with significant weight gain in all rats by 90 days. PA and PA+G implanted rats gained significantly more weight at 90 days compared to the other mesh types and the control, suggesting that these were better tolerated by the animals.

In line with previous studies examining several commercially available mesh types [21], [22], both our new meshes and the PP meshes showed contraction at all time points. In general, a correlation can be drawn between collagen deposition and mesh contraction for the PA+G, PEEK and PP mesh types, with an increase in collagen deposition leading to an increase in mesh contraction. It has been reported that both folding and contraction after implantation are responsible for side effects such as pain and tissue erosion [8]. No major differences in adhesion scores were determined between the mesh types assessed using the Lorenz adhesion scores [18]. PA had a significantly smaller adhesion area at 90 days compared to PEEK and PP, indicating that PA might produce fewer adhesions in the long term. No significant differences were found between the other mesh groups. Similar to our findings Boehm et al. compared Ultrapro® (combined PP and absorbable Monocryl®) and Prolene® (polypropylene) with no significant differences in terms of adhesions between the groups [21].

Uniaxial tensile testing was used to assess the biomechanical properties of explanted meshes, with stiffness represented by the slope of the load-elongation curve. Curves for the day 90 explants were found to be bilinear, with a region of initial low stiffness (toe region), followed by a region of higher stiffness (linear stiffness), as found by Afonso et al [23]. Biomechanical testing found all mesh types to be stiffer on day 90 compared to their day 7 counterparts, when loaded beyond strains of 4.2%. Collagen levels decreased between days 7 and 90 for some mesh types, suggesting that collagen alone did not contribute to increased explant stiffness; nor did mesh contraction since contraction was similar for all mesh types except PP. The present results are likely due to a combination of innate mesh mechanical properties and the amount, type and orientation of collagen formed, as suggested by the relative differences in stiffness between the 90 day explanted meshes and collagen levels. Separate tensile studies conducted on these meshes gave no indication of mesh creep, and without a necrotic response, it seems unlikely that any plasticizing material in the polymer was released. The large quantity of collagen present on the PA+G meshes at all time points, except day 60 (for which low rat weights were recorded), suggests that the gelatin coating promoted collagen deposition, or was associated with a cellular response that promoted an environment for fibroblast infiltration, collagen secretion and tissue remodelling. When the gelatin coating had almost fully degraded by day 90 (Fig. S2C), mesh pores became accessible to cells (Fig. S2B h), and permitted collagen synthesis, particularly within the large pores, between the filament bundles. Generally, the level of collagen decreased between 30 and 90 days for all mesh types. This outcome could be related to the fact that the meshes themselves do not promote tissue integration in the long term, or the type of tissue response is too rapid and does not allow for gradual cell infiltration, collagen synthesis and tissue remodelling that may be occurring with the PA+G meshes. We have addressed the need for a more objective analysis of collagen in mesh explants as identified in previous studies [17]. However it is difficult to make comparisons with these studies and the current quantitative evaluation. Our novel image analysis enables quantification of the amount of collagen and its relationship to the mesh filaments, whereas other studies have scored the organisation of collagen fibres which is also important for mechanical strength [16], [24]. Irrespective of this, collagen levels diminish, where in fact, collagen remained more closely associated with mesh filaments, rather than encapsulating the entire implant. In addition, the Sirius red stain does not take into account packing density of collagen fibrils or type of collagen around the mesh filaments, both of which will impact on the mechanical behaviour of the tissue.

Neovascularisation as measured by CD31 immunostaining reached highest levels at 60 and 90 days which is in agreement with previous studies for PP meshes [7], [17]. In the current study, PP meshes were consistently less vascularised over the entire time period compared with all new mesh types. In particular, PA and PEEK meshes were superior to PP meshes at 60 and 90 days, perhaps indicating these materials are intrinsically more permissive for angiogenic processes, or are simply comparable to vascularisation in control tissues, and the PP meshes actually inhibit the process. Vascularisation could be further improved by incorporating angiogenic factors onto the mesh filaments during fabrication [25].

Smooth muscle cells and myofibroblasts are cells implicated in wound healing where strengthening wound tissue is important. Our studies show a steady increase in alpha smooth muscle actin until 60 days. At this time point, all 3 new meshes were associated with significantly elevated smooth muscle cells compared with the PP meshes. These cells are finally lost through apoptosis after healing is complete explaining the significant decrease at 90 days. Acquisition of a smooth muscle cell coat by neovessels increases their stability, a desirable feature in the repair of tissues, including those that have herniated, by providing a stable blood supply to nourish neotissue and maintain its integrity in the long term.

In addition to the novelty of the new types of polymer meshes and biological coating used in this study, the strength of the current study is reflected in the use of computer-based analysis of the immunohistochemically stained slides using image analysis software. Histological analysis is the most commonly used technique in assessing new meshes [9]. Qualitative and semi-quantitative analysis of tissue by histology/immunohistochemistry has its limitations. Apart from the fact that different parts of the meshes are studied for histology making comparisons difficult [26], [27] more relevant is the problem of subjectivity during the scoring process. In the current study, subjective bias during the scoring process is excluded as the software detects the positive signal in each immunostained image on the same set of 4 centrally placed mesh filaments and calculates the percentage stained area. Also, for the first time, we report a significant gradient for inflammatory cells surrounding mesh filaments, by using the software to examine staining and cell numbers within 50 µm increments from the surface of the filament bundles. Using the image analysis software allowed us to investigate the same area of stained tissue for every image and precisely focus on the area of interest.

A limitation of this study is the choice of model for assessing *in vivo* biocompatibility of mesh to be used for fascial defect repair. The rat abdominal wall hernia model does not mimic the clinical environment. However, this model is well accepted for initial testing of newly designed meshes for both synthetic and biological materials in hernia repair, including pelvic organ prolapse [28]. The clinical interpretation needs to be carefully considered in animals lacking any pathological conditions.

The optimal mesh for hernia or POP repair has not been developed yet [8]. Our ongoing studies indicate that PA+G meshes promote good cell attachment and proliferation for mesenchymal stem cell growth and may be suitable as meshes, (data not published). In conclusion, the new mesh types investigated in this study might form the basis of a new option for the future treatment of fascial defects.

## Supporting Information

Figure S1 **Schematic of overlay technique.** A: Ventral full-thickness abdominal defect involving the transversalis fascia, rectus abdominis muscle, and peritoneum. Meshes (dotted green line), in direct contact with the viscera and skin were sutured with slight overlay to the abdominal wall, B: Rat incisional hernia model in control groups. The defect (dotted red lines) was repaired by manipulating the contralateral full-thickness abdominal wall (blue line). Photographs of rat abdominal wall C. At time of mesh implantation (PA) B. At 7 days of mesh implantation (PA+G) C. At 90 days of mesh implantation (PA+G) representative for all mesh groups.
(TIF)

Figure S2 **H+E stained sections from rats implanted with synthetic mesh.** A. Composite picture showing typical distribution of mesh filament bundles of PA at 7 days. B. Composite picture with several mesh filaments of PA at 90 days. C. Single filament bundles at higher power for control, PA, PA+G, PEEK and PP mesh after 7 days and. 90 days implantation. * denotes gelatin around PA filaments.
(TIF)

Figure S3
**Biomechanical properties of explanted mesh types and control on day 7 (- - - lines) and day 90 (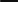 lines).** Explant stiffness is indicated by the slope of the curve, with stiffer materials possessing a steeper gradient. This is the same data shown in Figure 3 (means) and in addition includes 95% CI error bars.(TIF)

Figure S4 **Gradient analysis around the mesh filaments in 50 µm increments in PEEK meshes (representative example for all meshes).** A. CD68 positive staining at 7, 30, 60 and 90 days. *: p<0.05, ***: p<0.001. B. αSMA staining at 7, 30, 60 and 90 days. *: p<0.05, ***: p<0.001
(TIF)
