# Supplementary figures and images for: A Preclinical Evaluation of Alternative Synthetic Biomaterials for Fascial Defect Repair Using a Rat Abdominal Hernia Model
Source: PLoS One. 2012 Nov 20;7(11):e50044. doi: 10.1371/journal.pone.0050044 (PMC3502256; doi:10.1371/journal.pone.0050044)

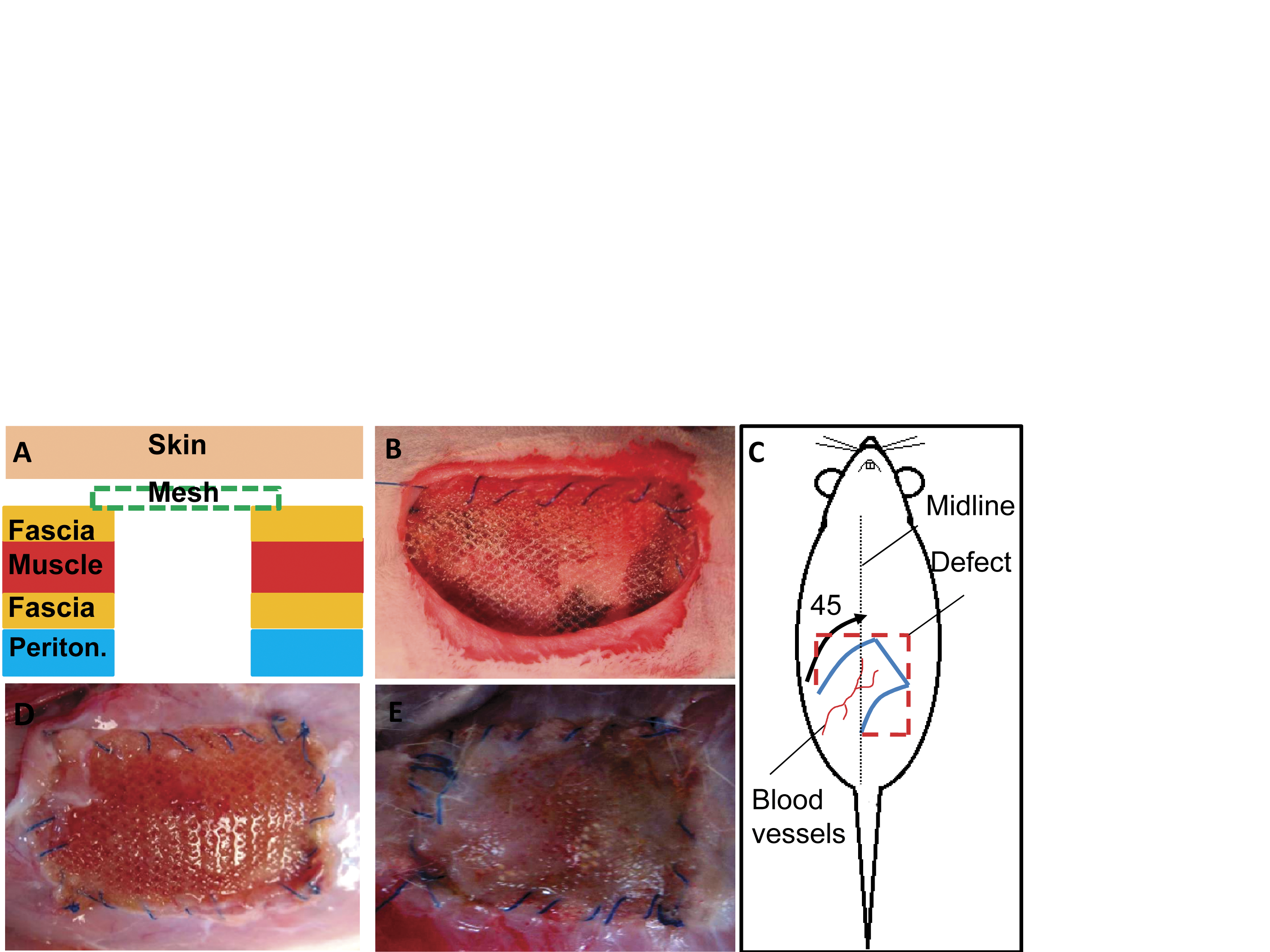

Supplement: Figure S1 — Schematic of overlay technique. A: Ventral full-thickness abdominal defect involving the transversalis fascia, rectus abdominis muscle, and peritoneum. Meshes (dotted green line), in direct contact with the viscera and skin were sutured with slight overlay to the abdominal wall, B: Rat incisional hernia model in control groups. The defect (dotted red lines) was repaired by manipulating the contralateral full-thickness abdominal wall (blue line). Photographs of rat abdominal wall C. At time of mesh implantation (PA) B. At 7 days of mesh implantation (PA+G) C. At 90 days of mesh implantation (PA+G) representative for all mesh groups. (TIF) [file pone.0050044.s001.tif]

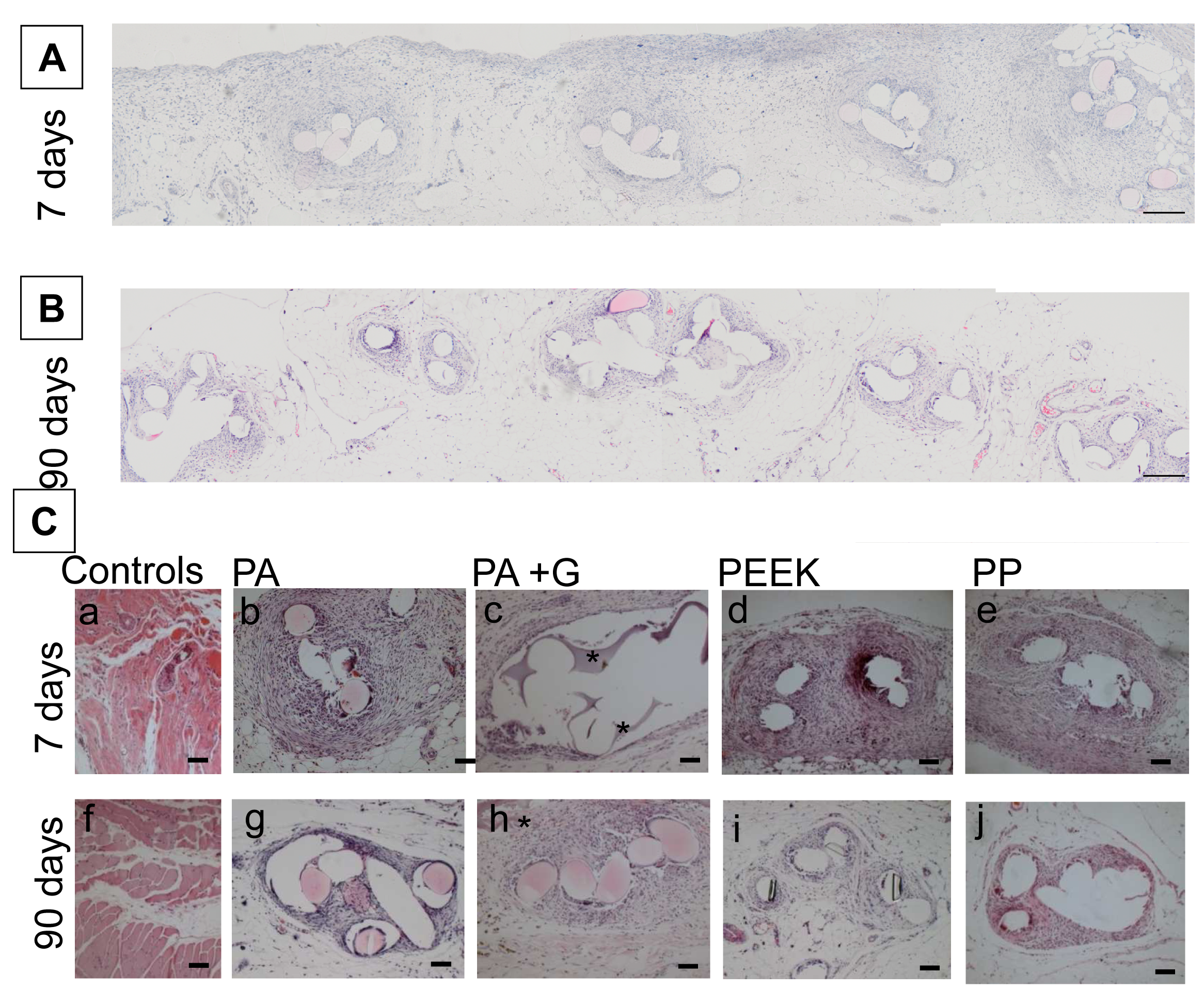

Supplement: Figure S2 — H+E stained sections from rats implanted with synthetic mesh. A. Composite picture showing typical distribution of mesh filament bundles of PA at 7 days. B. Composite picture with several mesh filaments of PA at 90 days. C. Single filament bundles at higher power for control, PA, PA+G, PEEK and PP mesh after 7 days and. 90 days implantation. * denotes gelatin around PA filaments. (TIF) [file pone.0050044.s002.tif]

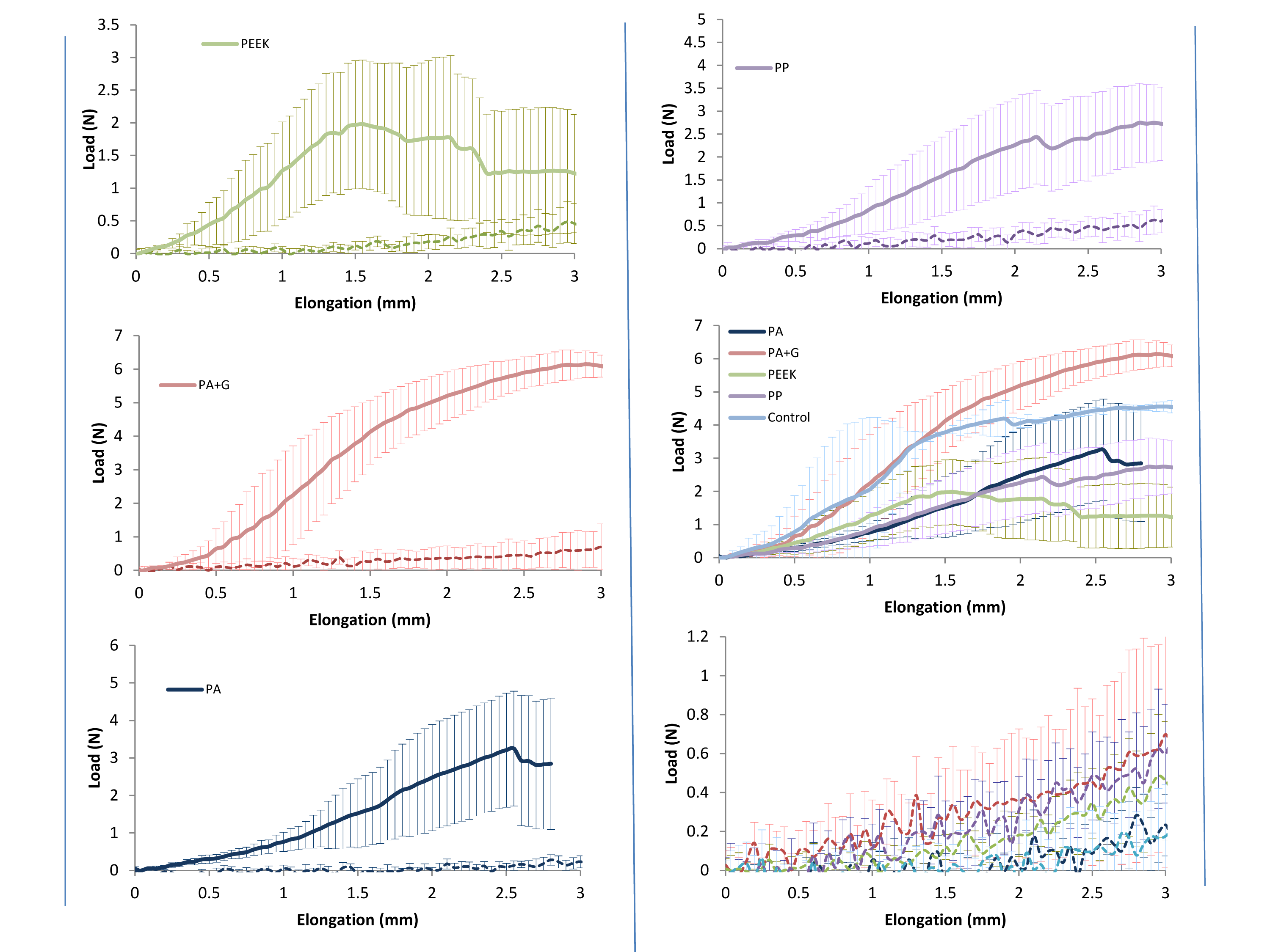

Supplement: Figure S3 — Biomechanical properties of explanted mesh types and control on day 7 (- - - lines) and day 90 ( lines). Explant stiffness is indicated by the slope of the curve, with stiffer materials possessing a steeper gradient. This is the same data shown in Figure 3 (means) and in addition includes 95% CI error bars. (TIF) [file pone.0050044.s003.tif]

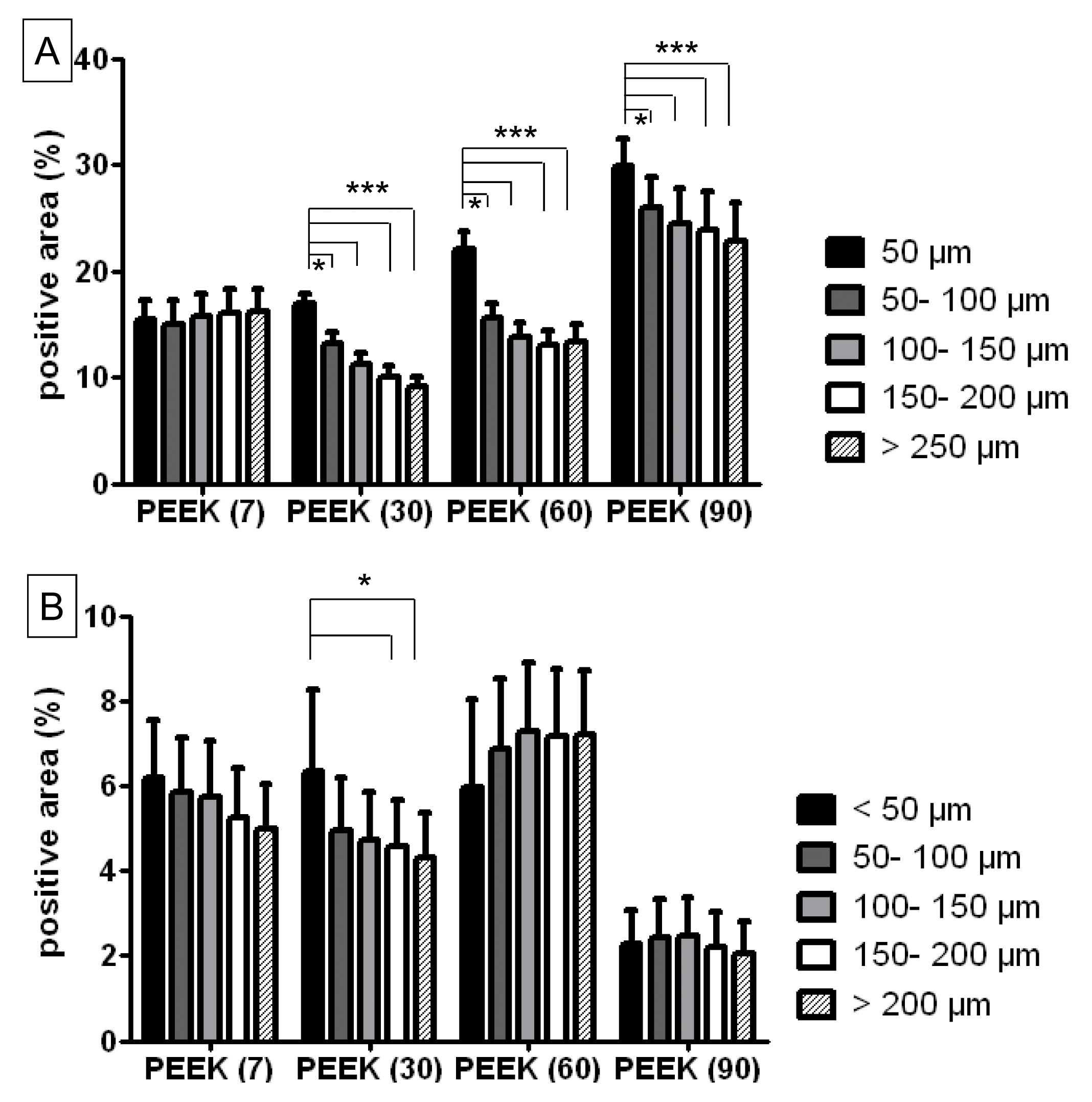

Supplement: Figure S4 — Gradient analysis around the mesh filaments in 50 µm increments in PEEK meshes (representative example for all meshes). A. CD68 positive staining at 7, 30, 60 and 90 days. *: p<0.05, ***: p<0.001. B. αSMA staining at 7, 30, 60 and 90 days. *: p<0.05, ***: p<0.001 (TIF) [file pone.0050044.s004.tif]
